# Supplementary material for: Deep learning predicts postsurgical recurrence of hepatocellular carcinoma from digital histopathologic images
Source: Sci Rep. 2021 Jan 21;11:2047. doi: 10.1038/s41598-021-81506-y (PMC7820423; doi:10.1038/s41598-021-81506-y)
Supplement: Supplementary file 6 — Supplementary Figures. [file 41598_2021_81506_MOESM6_ESM.pdf]

Deep learning predicts postsurgical recurrence of hepatocellular carcinoma from digital histopathologic images

Rikiya Yamashita[1,2], Jin Long[2], Atif Saleem[3], Daniel L. Rubin[1,2§], Jeanne Shen[2, 3§*]

[1] Department of Biomedical Data Science, Stanford University School of Medicine, 1265 Welch Road, Stanford, CA 94305, USA

[2] Center for Artificial Intelligence in Medicine and Imaging, Stanford University, 1701 Page Mill Road, Palo Alto, CA 94304, USA

[3] Department of Pathology, Stanford University School of Medicine, 300 Pasteur Drive, Stanford, CA 94305, USA

[§] *Equal contribution*

[*] Address correspondence to:

Dr. Jeanne Shen

Department of Pathology

300 Pasteur Drive, L235, Stanford, CA 94305-5324

Email: jeannes@stanford.edu

Supplementary Figures

Supplementary Figure 1: Histograms of HCC-SurvNet Risk Scores

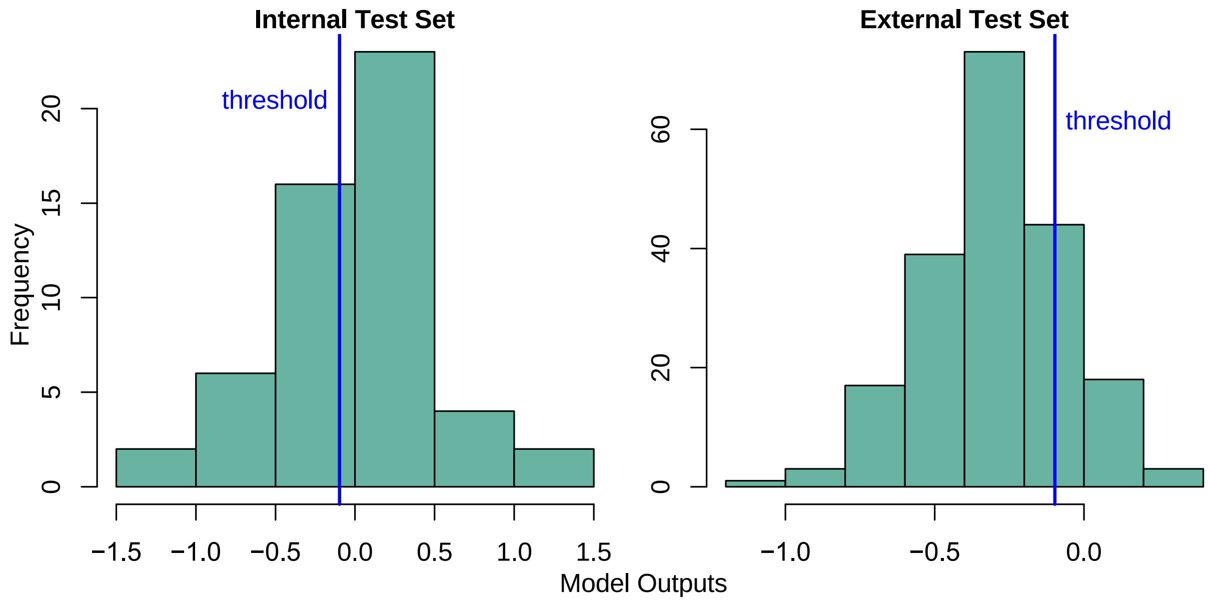

The histograms show the distributions of HCC-SurvNet's risk scores within the internal (left) and external (external) test sets separately. A threshold used for patient stratification into low- and high-risk groups, which was determined on the validation set from TCGA-HCC, is visualized as a blue vertical line (threshold = -0.0978).

Supplementary Figure 2: Univariable Cox Proportional Hazards Regression Analysis with Restricted Cubic Splines

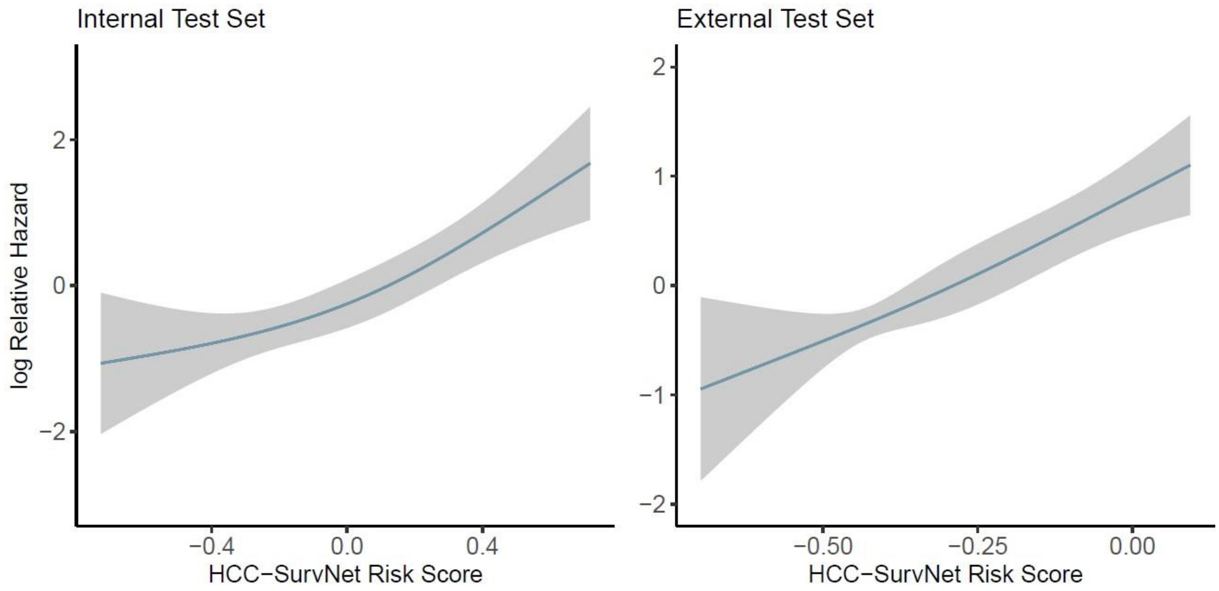

A continuous linear association between HCC-SurvNet's risk score and the log relative hazard for RFI was observed upon analysis of the internal test set (left), and even more significantly for the external test set (right). The blue line represents the fitted line of the association between HCC-SurvNet's risk score and the log relative hazard for RFI; the shaded region represents the 95% CI.

Abbreviations: CI, confidence interval; RFI, recurrence-free interval
